# Long-Distance Effects of Insertional Mutagenesis

**DOI:** 10.1371/journal.pone.0015832

**Published:** 2011-01-05

**Authors:** Ruchi Singhal, Xiaotao Deng, Alex A. Chenchik, Eugene S. Kandel

**Affiliations:** 1 Department of Cell Stress Biology, Roswell Park Cancer Institute, Buffalo, New York, United States of America; 2 Cellecta, Inc., Mountain View, California, United States of America; University of South Florida College of Medicine, United States of America

## Abstract

**Background:**

Most common systems of genetic engineering of mammalian cells are associated with insertional mutagenesis of the modified cells. Insertional mutagenesis is also a popular approach to generate random alterations for gene discovery projects. A better understanding of the interaction of the structural elements within an insertional mutagen and the ability of such elements to influence host genes at various distances away from the insertion site is a matter of considerable practical importance.

**Methodology/Principal Findings:**

We observed that, in the context of a lentiviral construct, a transcript, which is initiated at an internal CMV promoter/enhancer region and incorporates a splice donor site, is able to extend past a collinear viral LTR and trap exons of host genes, while the polyadenylation signal, which is naturally present in the LTR, is spliced out. Unexpectedly, when a vector, which utilizes this phenomenon, was used to produce mutants with elevated activity of NF-κB, we found mutants, which owed their phenotype to the effect of the insert on a gene located tens or even hundreds of kilobases away from the insertion site. This effect did not result from a CMV-driven transcript, but was sensitive to functional suppression of the insert. Interestingly, despite the long-distance effect, expression of loci most closely positioned to the insert appeared unaffected.

**Conclusions/Significance:**

We concluded that a polyadenylation signal in a retroviral LTR, when occurring within an intron, is an inefficient barrier against the formation of a hybrid transcript, and that a vector containing a strong enhancer may selectively affect the function of genes far away from its insertion site. These phenomena have to be considered when experimental or therapeutic transduction is performed. In particular, the long-distance effects of insertional mutagenesis bring into question the relevance of the lists of disease-associated retroviral integration targets, which did not undergo functional validation.

## Introduction

Insertional mutagenesis is a modification of target DNA via incorporation of additional bases. Insertion of long DNA fragments naturally happens during retroviral infection and transposition of mobile elements. It is also a byproduct of some common techniques of genetic engineering and gene therapy. In the latter case, it has been implicated as the cause of therapy-associated malignancies [1], and serendipitous activation of growth-promoting genes by insertion of a retroviral vector may be responsible for successful expansion of genetically engineered cells in gene therapy patients[2].

Importantly, insertional mutagenesis could be used to generate pools of randomly genetically-altered cells or organisms for forward genetics applications. In this case, the mutants with a phenotype of interest are selected, and the genetic loci tagged by inserts in such mutants are further investigated as candidate regulators of the mutant phenotype. For example, the genes at the sites of retroviral insertions in mouse tumors are treated as likely oncogenes or tumor suppressors[3].The yield of phenotypically-detectable mutants is greatly increased when the inserted fragment carries a strong promoter, which drives transcription of the adjacent host DNA, and such mutants could be distinguished from the spontaneous ones by virtue of their dependence on the promoter function [4]. Overall, insertional mutagenesis provides an efficient, cost-effective, unbiased and broadly applicable functional approach to identification of regulators of various biological processes (discussed elsewhere [4], [5]).

In our prior work [4], we relied on retroviral vectors for efficient delivery of a mutagenic regulated promoter cassette as a means of generating insertional mutants for gene discovery studies. This was achieved by placing the regulated promoter internally in a self-inactivating virus backbone. While the LTRs in such a vector are transcriptionally inactive following integration, they still retain the original polyadenylation site, which plays an important role in preserving the defined structure of the viral transcript. The presence of the polyadenylation site in the retroviral LTR creates an apparent problem for the production of fusion transcripts: one may expect the outbound transcript to be cut and polyadenylated prior to reaching the host DNA. Possible solutions to this problem include orienting the internal outbound promoter opposite to the retroviral LTRs [4], which may create a problem during production of the virus; removing the polyadenylation signal from the LTRs [6], which requires very extensive modification of the vector backbone and may facilitate additional structural changes during viral replication; or giving up the use of retroviral backbones in favor of other vectors, such as DNA-based transposons [7], [8].

However, there are some indications that the problem may be not so serious. First, there are evidence that at least some retroviral LTRs permit a considerable amount of read-through transcription [9]. Second, there are reports that polyadenylation signals within introns could be co-transcriptionally removed and thus rendered inactive by splicing machinery [10], [11]. Therefore, if transcription continues through the LTR, and the splice donor site ends up matched with an appropriate splice acceptor, then the polyadenylation signal of the LTR could be lost during splicing, and a stable hybrid transcript encompassing both the vector-and host-derived sequences could be formed. In the present report, we describe our experience with a vector design that relies on this phenomenon and the effect that the elements of this construct have on genes at a considerable distance from the integration site.

## Results

### HIV LTR is inefficient in preventing splicing of vector- and host-derived sequences

**Figure 1:**
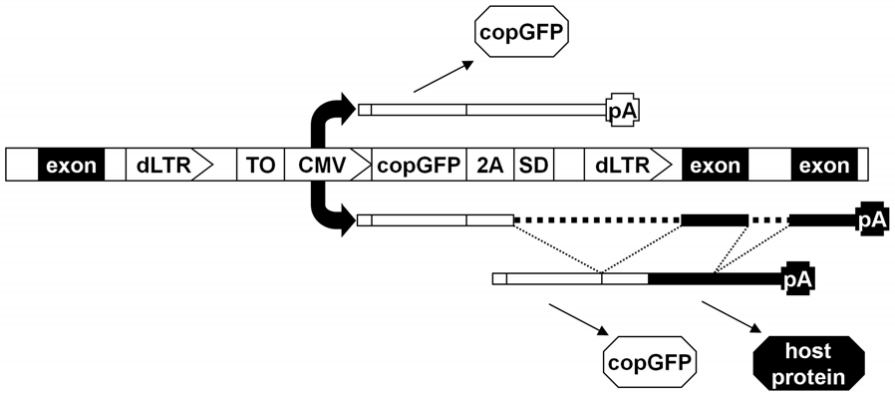
Functional organization of pAIM. The vector is shown in the form of a provirus collinearly integrated in a host gene. The vector is flanked by promoter-deficient LTRs (“dLTR”), which harbor Cre-recombinase recognition sites. The immediate early promoter and enhancer region from human cytomegalovirus (“CMV”) is preceded by a cluster of tet-operators (“TO”), which permit suppression of this promoter in the presence of a tetracycline-controlled transcription silencer (tTS). The coding region of copGFP (“copGFP”) ends with the 2A sequence and an unpaired splice donor (“2A” and “SD” respectively). In the scenario shown above the vector, CMV-driven transcript is polyadenylated on the LTR-derived sequence and encodes only copGFP. In the scenario shown below the vector, transcription continues into the host DNA, and a mature fusion product is produced by splicing, which removes the LTR sequences. If the open reading frames of copGFP and the host gene coincide, both copGFP and a host protein could be produced when the RNA is translated.

To test the prediction that successful pairing of an internal splice donor site with a host splice acceptor may result in removal of the LTR-encoded polyadenylation signal, we constructed a vector (designated pAIM) based on an HIV-1 backbone (Figure 1) that should be suitable for reversible insertional mutagenesis. In the plasmid form, the 5′-LTR is promoter-competent, while the 3′-LTR is promoter-deficient. During the natural replication cycle, both LTRs become identical, carrying the inactivating deletion in the promoter. The presence of Cre recombinase recognition sites (loxP) in the LTR permits excision of the promoter cassette. An internal CMV promoter is collinear with the viral genome. It is followed by the coding region of *Pontellina plumata* GFP (copGFP) [12] and an unpaired splice donor site. The region of copGFP ends with a sequence that codes for picornaviral 2A peptide. When encountering this sequence, ribosome is known to proceed without forming a peptide bond. Consequently, two physically separate peptides could be created from a single open reading frame [13]. If splicing is successful, host exons and, eventually, polyadenylation signals will be linked to the vector-encoded fragment, and a host-encoded peptide may be produced. CopGFP, expressed either from a vector-encoded or a hybrid transcript, is expected to mark all transduced cells whenever CMV promoter us functional. The CMV promoter is also preceded by an array of tetracycline operators. This feature could be used to reversibly target the promoter for inactivation using tTS, a tetracycline-controlled transcription silencer constructed by fusing the tetracycline repressor protein (TetR) with a KRAB silencing domain (SD^Kid-1^) [14].

The vector was used to infect a culture of immortalized mouse embryonic fibroblasts at a low infection rate (<10%; judged by the incidence of copGFP-expressing cells), and individual clones were established. Ten clones that displayed green fluorescence were chosen for further analysis. Quantitative PCR on their genomic DNA confirmed single-copy integration (data not shown). We relied on nested ligation-mediated PCR on the cDNA from these clones to look for the presence of hybrid transcripts. Prior to adapter ligation, the cDNA was digested with frequently cutting enzyme MboI. Due to the presence of an MboI site in the vector between the splice donor and the 3′-LTR, the unspliced transcript is expected to produce a distinct band, while bands of variable sizes are expected from the spliced fusion transcripts. Importantly, out of the ten clones, at least four demonstrated prominent expression of putative fusion products.

**Figure 2:**
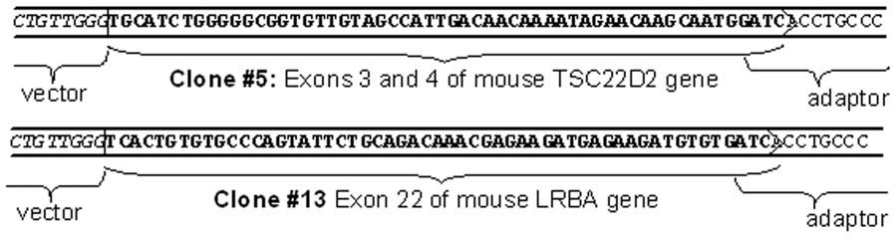
Identification of the fusion transcripts generated by trans-LTR splicing in two independent clones. The products of nested ligation-mediated PCR on MboI-digested cDNA were sequenced. As predicted, the clones contain host sequence, followed by an MboI site (GATC) and the adapter. The 5′-end of the transcript is derived from the vector. The junction point corresponds to host intron/exon boundary.

The PCR products corresponding to the four putative fusion transcripts were sequenced, confirming the presence of both the host- and vector-derived sequences. Two of the fragments were unambiguously mapped to unique sequences in mouse genome and contained precise fusion of the vector sequences to exons of mouse TSC22D2 and LRBA genes (Figure 2). The precise maintenance of the exon boundaries confirms splicing as the mechanism responsible for the production of the hybrid RNAs. We concluded that in our construct the combination of read-through transcription and splicing may alleviate the potential hurdle of polyadenylation site in the modified HIV-1 LTR.

### Long-distance transactivation of RIPK1 gene by insertional mutagenesis

**Figure 3:**
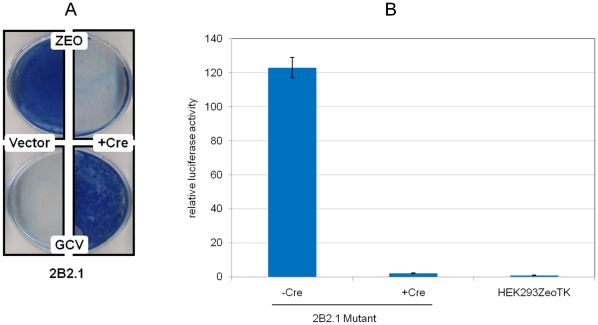
Reversible activation of NF-κB in mutant cells. (A) Reversible pattern of drug resistance in the mutant clone. Mutant clone 2B2.1 was cultured in Zeo (upper panels) or GCV (lower panels) after infection with Cre (right panels) or the empty vector (left panels). 2.5×10^5^ cells per 6 cm plate were treated for a week, and viable cells were visualized by methanol fixation and methylene blue staining. Similar results were observed in clones 2B1.4 and 2B3. (B) Reversible up-regulation of an NF-κB reporter in the mutant cells. Luciferase reporter assay was used to compare the activity of NF-κB in 2B2.1 cells transduced with either Cre or an empty vector to that in the parental HEK293ZeoTK cell line. The activity of a transiently transfected luciferase reporter was normalized for that of co-transfected constitutive beta-galactosidase expression construct. The data is presented in relative units, with the normalized activity of the reporter in the parental cell line (HEK293ZeoTK) taken as 1. Mutant clones 2B1.4 and 2B3 demonstrated a similar pattern.

HEK293ZeoTK is a cell line derived from HEK293 that carries Zeocin-resistance marker and HSV-1 thymidine kinase gene as two transgenes, each under the control of an NF-κB-depended E-selectin promoter [15]. The cells represent a convenient system to screen mutants with alterations in NF-κB pathway: the cells with active NF-κB could be selected in the presence of Zeocin, or could be selectively eliminated in the presence of ganciclovir. In this manner, numerous mutants with either increased or decreased activity of this pathway have been selected [6], [7], [8], [15], [16], [17]. Infection of these cells with the construct described in Figure 1 resulted in multiple Zeocin-resistant clones. As expected, the mutants had constitutively elevated activity of NF-κB, and this activity, as well as the pattern of Zeocin and ganciclovir resistance, was readily reverted when the inserted cassettes were removed by site-specific recombination or shut-down by expression of the tet-repressor protein (see Figure 3A and Figure 3B for examples). The observations allowed us to attribute the mutant phenotypes to the activity of the inserted fragment.

**Figure 4:**
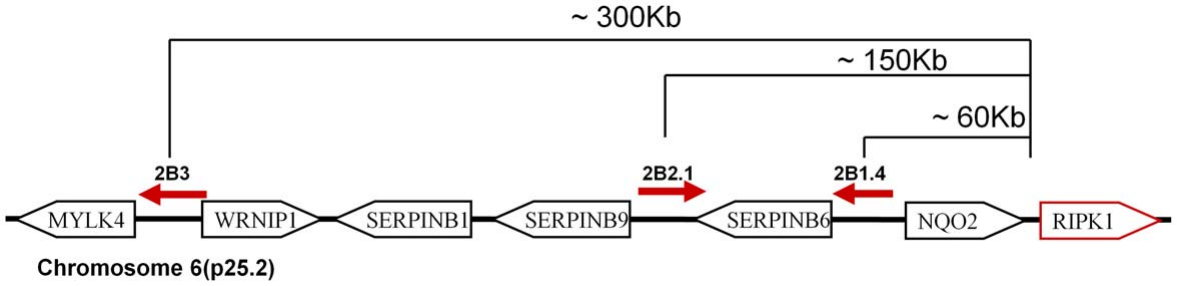
Location of inserts in the three mutant clones. A fragment of p25 region of chromosome 6 is shown, indicating the relative positions of known genes. The inserts are shown as arrows pointing in the direction of the CMV promoter. Approximate distances from the first annotated exon of RIPK1 are indicated. The drawing in not to scale.

**Figure 5:**
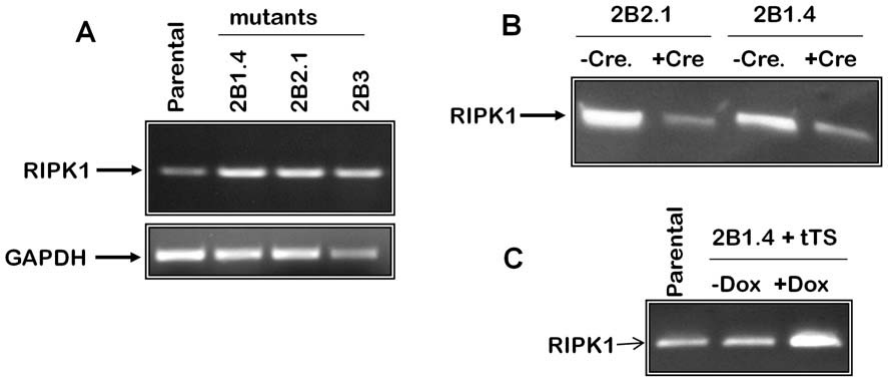
Examples of regulated expression of RIPK1 in mutant cells. (A) Elevated expression of RIK1 in the mutant clones. RNA was isolated from mutant clones 2B2.1, 2B1.4 and 2B3, and from the parental HEK293ZeoTK cells, and analyzed by RT-PCR with RIPK1-specific primers (upper panel). The bottom panel presents results of RT-PCR on the same samples with primers for a housekeeping gene (GAPDH). (B) Dependence of RIPK1 expression in the mutant clones on the presence of the insert. Mutant clones 2B1.2 and 2B1.4 were infected with a Cre-expressing construct or the respective empty vector control. The cell lysates were compared by Western blotting with polyclonal anti-RIPK1. (C) Dependence of RIPK1 expression in the mutant clones on the function of the inserted promoter. Mutant clone 2B1.4 infected with a tTS-expressing construct was cultured with or without doxycycline. The cell lysates were analyzed by Western blotting with polyclonal anti-RIPK1 and compared to those of the parental HEK293ZeoTK cells.

**Figure 6:**
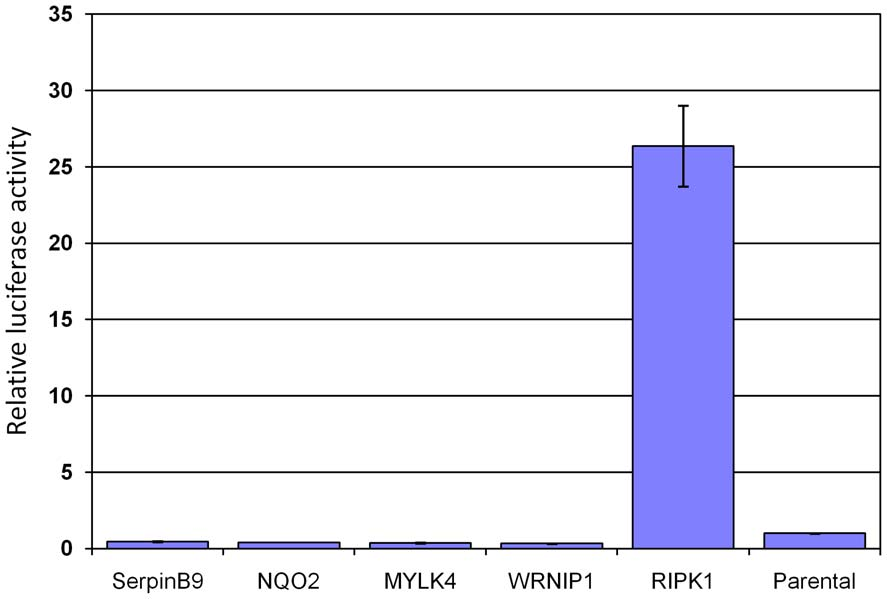
Activity of NF-κB upon overexpression of genes in the vicinity of inserts compared to parental HEK293ZeoTK cells. NF-êB dependent luciferase reporter activity was measured after cells were co-transfected with respective expression constructs. Only RIPK1 showed elevated NF-κB activity.

Three of the mutant clones (2B2.1, 2B1.4 and 2B3) attracted our interest: in each of them a single insert was located in p25 region of chromosome 6. The three mutants behaved identically in all the tests. Interestingly, RIPK1 gene, which codes for a known modulator of NF-κB signaling, also maps to 6p25, and mutations affecting RIPK1 were previously identified in similar screens[7]. However, the inserts in the current study were located considerably further upstream of any known RIPK1 exon. In fact, the current annotation of the human genome places several other genes in the close vicinity of the inserts (Figure 4). Nevertheless, RIPK1 expression was elevated in the mutant clones at the level of RNA and protein (Figure 5). In contrast, expression of the products of the neighboring genes, including those closest to the inserts, was undetectable or unchanged, and their forced overexpression in naïve cells failed to affect NF-κB activity (Figure 6 and data not shown). Importantly, RIPK1 expression was reduced in the mutants upon physical removal or transcriptional shut-down of the inserted cassette. The latter observation indicates that the effect is dues to the activity of the inserted fragment, rather than by destruction of some negative control elements. Interestingly, the insert orientation differed between the mutants. Thus, it is highly unlikely that the mutant phenotype could be accounted for by a vector-derived transcript fusing with the RIPK1 sequences, and our attempts at detecting such a fusion transcript were unsuccessful (data not shown).

**Figure 7:**
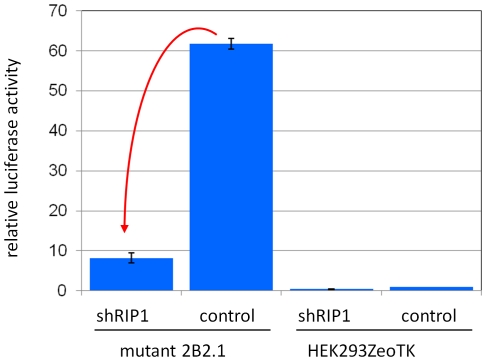
A example of the dependence of NF-κB activity in mutant cells on the function of RIPK1. Mutant 2B2.1 and parental HEK293ZeoTK cells were assayed for NF-κB activity using transient transfection of a luciferase reporter. A decline in the reporter activity (indicated by the red arrow) resulted from adding an shRNA against RIPK1 to the transfection mix. An shRNA against p53 (a protein that is expressed, but is inactivated in these cells) was used as the control.

In order to confirm that NF-κB activation in these cells is indeed dependent on the overexpression of RIPK1, we measured the activity of NF-κB-dependent luciferase reporter in the presence of shRNA directed against RIPK1. As before [7], in order to control for non-specific effects of RNA interference, we used an shRNA against p53, which is present, but inactivated in these cells. The example in Figure 7 demonstrates that interference with RIPK1, but not an unrelated protein (p53), reduced the activity of NF-κB in the mutants. Therefore, we concluded that the activity of the inserted cassette caused constitutive activation of NF-κB through up-regulation of RIPK1 expression without formation of a fusion transcript.

## Discussion

Various structural and biological features of retroviruses make retroviral vectors useful for studies the properties of mammalian transcription (e.g. [18]). In the described experiments, we observed that, despite the presence of a polyadenylation signal, the modified LTR of HIV-1 fails to efficiently prevent read-through transcription and accumulation of ensuing fusion transcripts. This is consistent with observations from other models, where splicing takes precedence over polyadenylation [10], [11]. It is worth noting that our observation on the efficiency of this process (4 out of 10 cases) is a conservative one: detection of the hybrid product was contingent upon the proximity of MboI site and the absence of secondary structures, which could potentially interfere with RT-PCR, in the hybrid RNA. Also, accumulation of the hybrid transcript, in addition to the removal of the LTR, might require trapping of a functional polyadenylation site in the downstream sequence.

Formation of hybrid RNAs via read-through is considered one of the major mechanisms of that generates transducing retroviruses [19]. The classical scheme [19] proposes formation of a read-through transcript, which originates at the 5′-LTR, incorporates an entire viral genome, and terminates in the adjacent host sequences. Such a transcript may be incorporated into the viral particle (albeit inefficiently, because of its size), but, due to the presence of an LTR in the middle of the RNA, has to undergo extensive re-arrangements during reverse transcription in order for a transducing virus to be formed. Our observations suggest that if internal sequences of a virus or a viral vector could act as splice donors, the 3′-LTR could be removed from such a transcript during splicing. The resulting RNA may retain the packaging signal, and could be converted into a transducing virus upon a single recombinational event during reverse transcription. This may be especially important for MLV-based vectors, which are often used without inactivation of the LTRs, and which may regain replication potential in the presence of a natural human retrovirus[20].

The fusion transcripts generated via splicing have an important difference from the ones generated via read-through alone: the host sequences are positioned much closer to the 5′-end of the RNA and, hence, are much more likely to be expressed, which could lead to undesirable consequences for experimental or therapeutic transduction. Consequently, the risk of a combination of read-through transcription and splicing has to be seriously considered when vectors are designed for such applications.

At this time we can only speculate about the molecular mechanism, which underlies the long-distance effect of the inserts on RIPK1 expression. Since the effect requires the function of the CMV fragment and is orientation-independent, it most resembles that of a classical enhancer. The question is how it can act at such distances and without significantly affecting the genes between the insertion site and *RIPK1*? One explanation is that it generates a widespread continuous signal (e.g. via chromatin modification) over hundreds of kilobases, but the promoters of intermediate genes are for some reason irresponsive to that particular stimulus (e.g. missing recognition sites for the factors that could sense it). Another explanation, which we find more plausible, is that the three-dimensional organization of chromosome 6 is such that the sites of integration are physically close to the beginning of *RIPK1* gene. This could occur via looping of a chromosome, a phenomenon, which was characterized in great detail, for example, in regard to the regulation of proto-oncogene Myc [21], [22]. Interestingly, structural aberrations or retroviral insertion sites that are located tens to hundreds of kilobases away from Myc gene are associated with elevated expression of this gene and are functionally equivalent to the ones occurring in direct proximity of that gene, but the possibility of their direct effect on Myc used to be dismissed based on the assumption that they are positioned too far and the effects of enhancers cannot “leapfrog” over long distances [23]. Of note, we failed to detect in the mutant cells expression of the putative shorter RIPK1 product (data not shown), which may originate from an internal promoter [7]. This is consistent with the notion that the upstream and the internal promoters are subject to distinct regulatory mechanisms.

Besides providing new insights into transcriptional regulation of *RIPK1*, our observations have important implications for the use of insertional mutagenesis in gene discovery. Insertional mutagenesis is typically conducted by insertion of a DNA fragment, which carries a complete potent viral promoter. A common approach would be to map a large number of insertion sites in the DNA from a pool of cells enriched for the phenotype of interest, and then argue that certain genes are significant for this phenotype because there are more commonly targeted in this pool than what may be predicted based on random integration. In most cases, publicly available lists of such genes are quite extensive and do not undergo individual validation [3]. An obvious drawback of this approach is that integration is known to be non-random, but there is no model to account for this bias, and, especially, for possible variation in this bias as a function of transcriptional state of individual loci and physiological state of the cell. On top of this, our experience suggests that even *bona fide* commonly targeted sites may be deceptive: the genes truly responsible for the phenotype could be located at a considerable distance away from the nominal integration target. Unlike Myc, which is positioned in a “gene desert” and may be considered the closest known gene even to relatively distant inserts, the phenomenon presented in this report takes place in a segment of a chromosome, which is packed with known or predicted genes. Thus, a purely bioinformatics approach would have misidentified the relevant gene. In view of this, we would like to argue that veracity of predictions unsupported by experimental validation cannot be taken for granted.

We have previously constructed insertional mutagens [4] that relied on the use of a minimal promoter controlled by binding sites of tetracycline transactivator protein (tTA), rather than an enhancer-containing fragment of CMV. Interestingly, the many mutants generated by those constructs were always attributable to the production of the fusion transcript, rather than an enhancer-like effect ([4] and unpublished data). This observation gives a hope that one may produce a more accurate gene-discovery system by carefully selecting a promoter that is included in the insertional mutagen.

Similar concerns are valid for inadvertent insertional mutagenesis during experimental and therapeutic transduction. Indeed, although some gene therapy patients have developed cancer following insertion of the therapeutic construct into or very close to LMO2 gene [24], another case was associated with an insertion 35 kbps upstream of (and in an opposite orientation to) the same proto-oncogene [25]. In this regard, vectors devoid of enhancer elements [26] may offer an attractive avenue for the design of safer gene-delivery vehicles.

## Materials and Methods

### Cell culture, drug treatment and viral transduction

All cells were cultured in Dulbecco's modified Eagle's medium with 10% FBS, and 1% penicillin and streptomycin at 37°C in the presence of 5% CO_2_. Zeocin and ganciclovir were used as described earlier [4]. Hygromycin (Roche) was used at 100 µg/ml.

All viruses were packaged in 293T cells (originally known as 293tsA1609neo[27]). Virus-containing media were collected at 48 h, filtered, supplemented with 5 ug/ml Polybrene, and applied to the target cells for an overnight incubation.

Prior to mutagenesis, HEK293ZeoTK cells were cultured in the presence of ganciclovir for about a week to remove any pre-existing mutants with activated NF-κB, and then infected with virus on 6 cm plate. Medium was changed after 24 h of infection. Next day the cells were replated into 15 cm plate, where they were treated with Zeocin for 12 days to select for the mutant clones.

### Plasmids

Full length RIPK1 expression construct, shRNA against RIPKI expressing plasmid and shRNA against p53 were as described [7]. pBabeHygro [28] was used a vector backbone for expression of Cre recombinase as described [3]. The cDNA for tetracycline-controlled transcription silencer (tTS), as well as the vector used for its expression (pLPCX) were from Clontech, Inc. All other constructs for full-lengths cDNA expression were purchased from Origene.

### Reporter assay and Western Blotting

NF-κB-dependent reporter plasmid (pE-selectin-luciferase) was transiently transfected using Lipofectamine Plus method (Invitrogen), and luciferase activity was measured and normalized for that of a co-transfected constitutively active â-galactosidase expression vector, as described earlier [4].

For the detection of RIPK1 protein, total cell lysates were prepared from 95% confluent plates and analyzed by the Western blotting using anti-RIPK1 primary antibodies (BD Transduction Laboratories), goat anti-mouse secondary antibodies (Sigma) and SuperSignal chemiluminescent substrate (Thermo Scientific).Chemiluminescence was recorded using FluoroChem HD2 camera (Alpha Innotech).

### PCR

The hybrid mRNA fragments were revealed using nested ligation-mediated PCR. The cDNA prepared from individual infected clones was digested with MboI and ligated to an MboI adapter (constructed by annealing of GATCACCTGCCC and CTAATACGACTCACTATAGGGCTCGAGCGGCCGCCCGGGCAGGT to enable the PCR suppression effect [29]). Subsequently, nested PCR was done using primers GTFwd1 (ACTCGGATAATACGACGCACGAGA) and AP1 (CCATCCTAATACGACTCACTATAGGGC) for the first stage, followed by the second stage using GTFwd2 (AAGGCTCAGGAGAGGGCAGAGGAA) and AP2 (ACTCACTATAGGGCTCGAGCGGC). The PCR products were separated on a gel, excised, and sequenced.

For insert mapping using inverse PCR, MspI- digested genomic DNA form the mutant clones were circularized by self-ligation, followed by nested PCR. The first round was conducted with HTOmspS1 (ACAGTGCAGGGAAAGAATAG) and HTOmspAS1 (ATGGTGAATTGATCCCATCTTG) primers; the second - with primers HTOmspS2 (TTCGTTGGGAGTGAATTAG) and HTOmspAS2 (CAGGGGAAAGAATAGTAGAC). The final PCR product was cloned into pCR8/GW/TOPOTA (Invitrogen) and sequenced.

RIPex1S (CTTCCTGGAGAGTGCAGAAC) and RIPex2AS2 (CTCCATCACCAGGGAGTACTTC) primers were used to assay RIPK1 mRNA expression by RT-PCR. A housekeeping gene (GAPDH) was used as an internal control (Forward: GGCTCTCCAGAACATCATCCCTGC, Reverse: GGGTGTCGCTGTTGAAGTCAGAGG).
